# Supplementary material for: Functional Analysis of Viable Circulating Tumor Cells from Triple-Negative Breast Cancer Patients Using TetherChip Technology
Source: Cells. 2023 Jul 26;12(15):1940. doi: 10.3390/cells12151940 (PMC10416943; doi:10.3390/cells12151940)
Supplement: Supplementary file 1 [file cells-12-01940-s001.zip › cells-2493962-supplementary.pdf]

S1

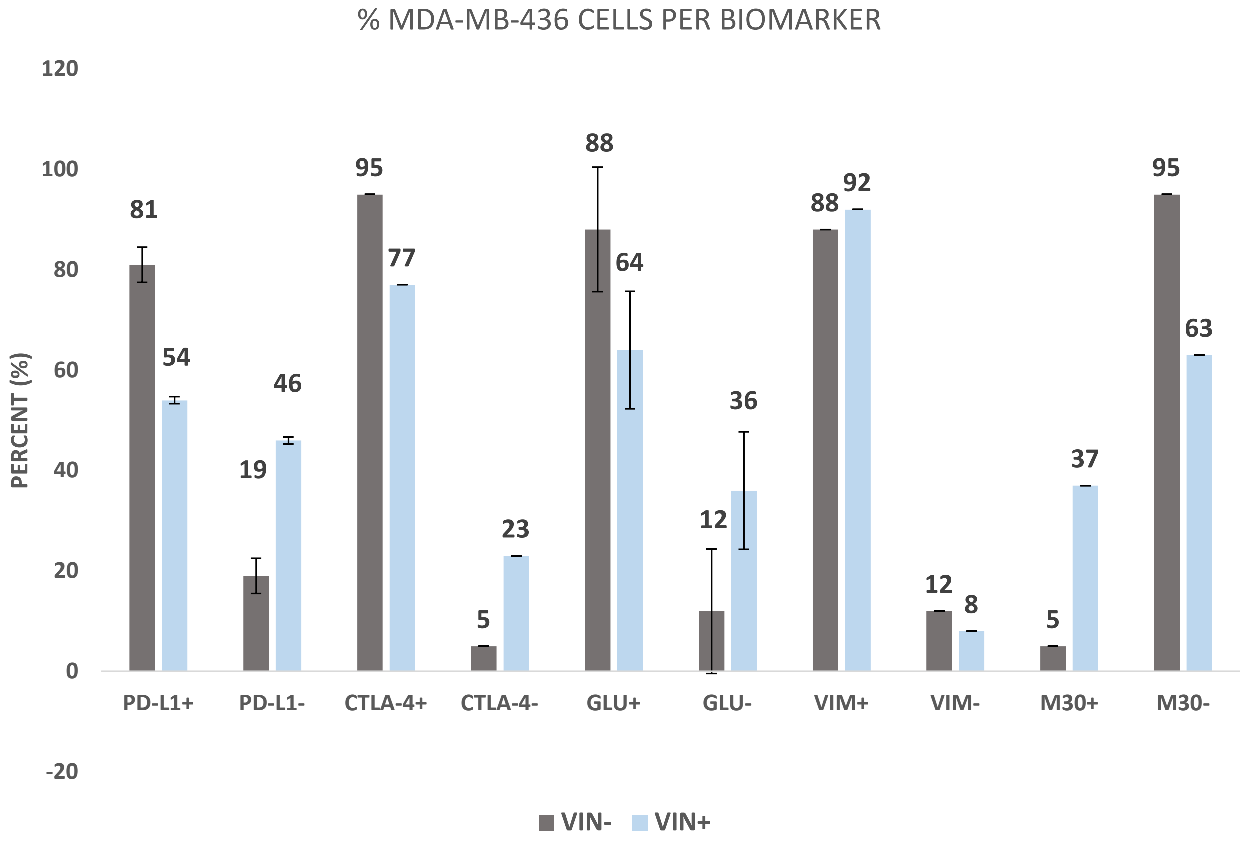

Figure S1: MDA-MB-436 cells in TetherChips. Percentage of MDA-MB-436 with the corresponding phenotypes before and after vinorelbine treatment (10 μM) for 1 h. Data are shown as mean ± SE.

S2

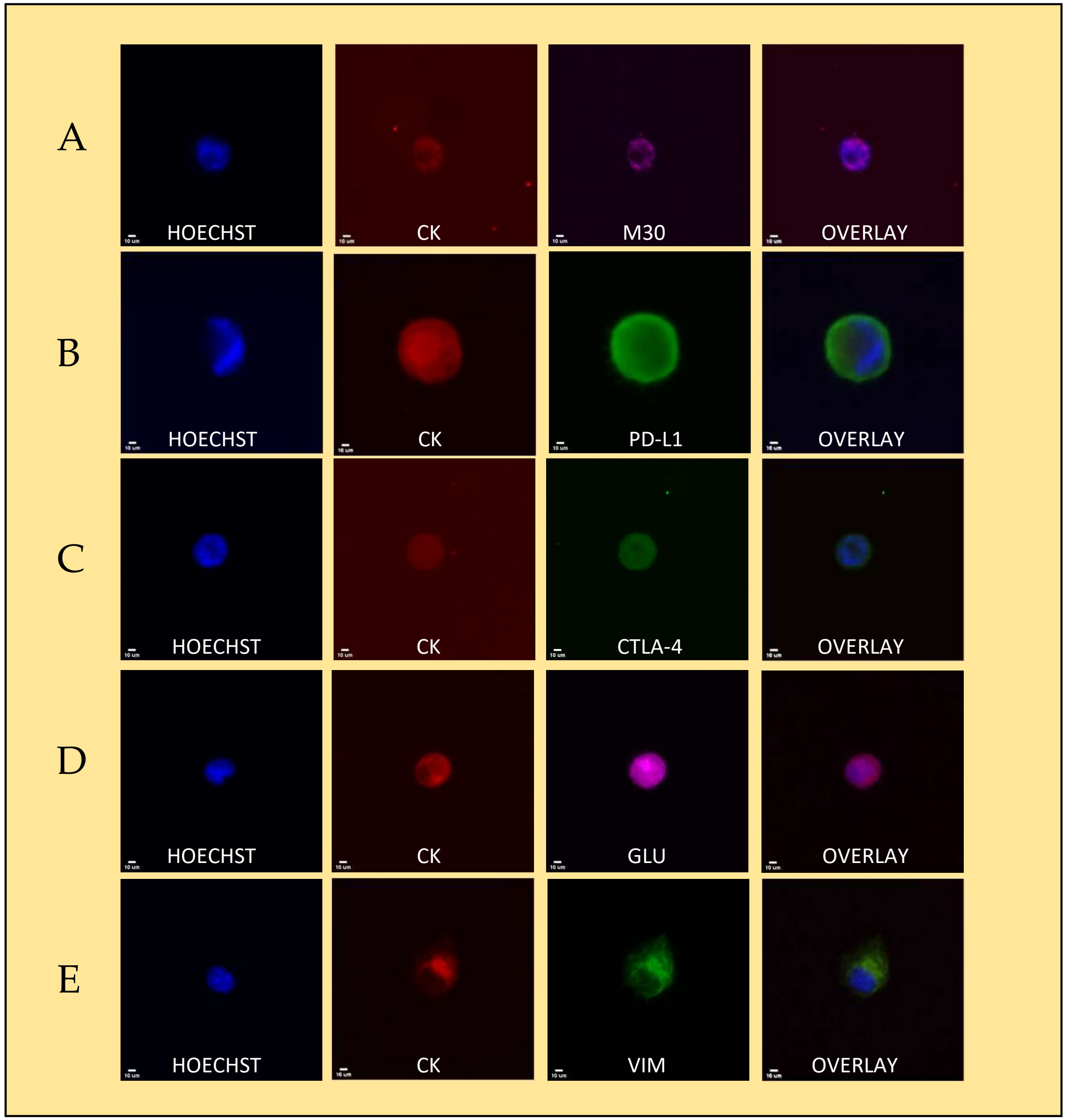

Figure S2: Expression of all the biomarkers in MDA-MB-436 cells. The first column represents nuclei stained with HOECHST; the second column represents cells expressing CK, the third cells expressing M30 CytoDeath, PD-L1, CTLA-4, GLU, and VIM; the fourth represents the overlay of the three channels. Representative panels of cells with (A) expression of M30 CytoDeath (apoptosis), (B) expression of PD-L1, (C) expression of CTLA-4, (D) expression of GLU, and (E) expression of VIM are shown. Micrographs were acquired from Vycap System. Controls were implemented adding 1000 cells in each well of the TetherChip. Scale bars = 10 µm.
